# Benign Mucinous Tumor of Ovary with a Sarcoma-Like Mural Nodule: A Case Report

**DOI:** 10.1155/2012/213765

**Published:** 2012-10-03

**Authors:** Sailabala Garikaparthi, Renuka Inuganti Venkata, Krishna Bharathi Yarlagadda, Annapurna Parvatala

**Affiliations:** Department of Pathology, Guntur Medical College, Andhra Pradesh, Guntur 522004, India

## Abstract

Sarcoma-like mural nodules occur predominantly in middle-aged women. Distinction of these lesions from true sarcomatous nodules and foci of anaplastic carcinoma is important because of the worse prognosis of these tumors in comparison with the favorable behavior of sarcoma-like mural nodules. In this report we describe the case of a 35-year-old woman with a mucinous ovarian tumor having a mural nodule in the wall.

## 1. Introduction

Mucinous cystic tumors of ovary whether benign or malignant may be associated with sarcoma-like mural nodules (SLMNs). These nodules resemble a gingival epulis or giant cell tumor of bone and are reactive rather than neoplastic. Their distinction from true sarcomatous mural nodules and foci of anaplastic carcinoma is extremely important since each of these lesions carries a different prognosis [1]. We describe a benign ovarian mucinous tumor with SLMN. The value of immunohistochemistry (IHC) in the differential diagnosis of these lesions is discussed.

## 2. Case Details

A 35-year-old female patient presented with lower abdominal pain for six months. On examination a swelling of 15 to 18 cms was detected. Ultrasonography revealed a right ovarian mass, and ovarian cystectomy was performed.

**Figure 1:**
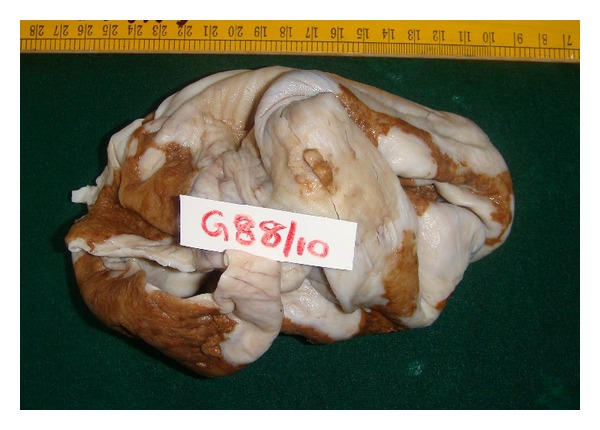
Ovarian tumor with mural nodule.

**Figure 2:**
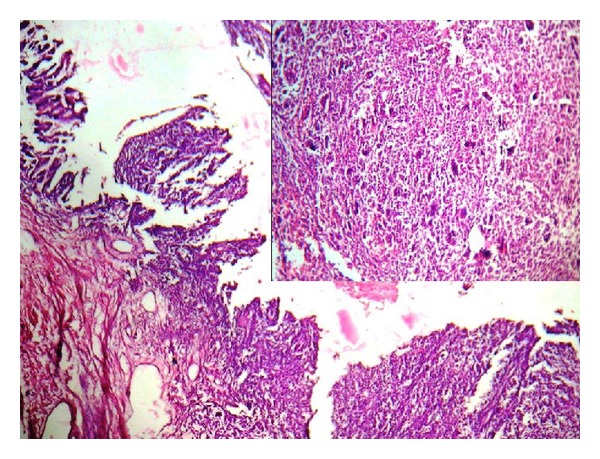
Microphotograph showing cyst lining and cellular mural nodule inset: SLMN with spindle-shaped stromal cells and giant cells (H and E ×100).

**Figure 3:**
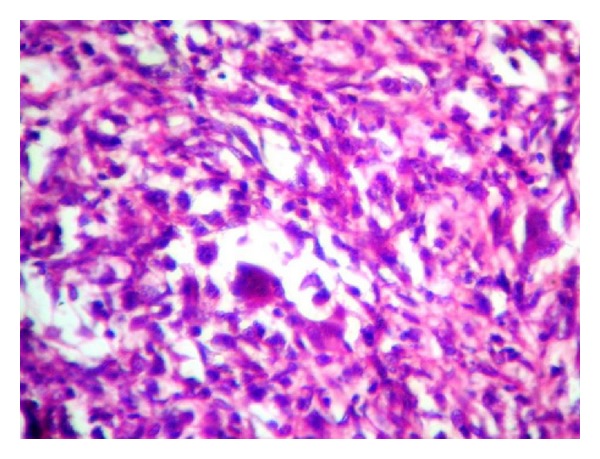
Osteoclastic type of giant cells and cellular stroma (H and E ×400).

Grossly the ovarian cyst measured 18 × 11 cms with an attached tube of 3 cms. The outer surface was whitish and smooth, with an elevation of 2 cms diameter (Figure 1). On cut section the cyst was unilocular with mucinous material and a gray-brown nodule. Microscopic examination showed features of a mucinous cystadenoma, surface epithelium being papillary and lined by tall columnar mucinous epithelium. Sections from the gray-brown nodule showed increased cellularity in the stroma with spindle- and polygonal-shaped cells, and plenty of osteoclastic type of giant cells (Figure 2). The stromal cells were monomorphic with no nuclear atypia, and minimal mitotic activity (1–5/10HPF) was seen. The giant cells were similar to osteoclastic giant cells of bone (Figure 3). Immunohistochemistry for Vimentin was positive, and pancytokeratin was only focally positive.

## 3. Discussion

Various types of mural nodules are found in the walls of mucinous ovarian tumors. They include anaplastic carcinoma, carcinosarcoma, and sarcoma-like nodules, mixed nodules, and leiomyomas [2]. We describe a benign ovarian tumor with a sarcoma like mural nodule (SLMN). Mucinous cystic tumors of ovary, whether benign, borderline, or malignant may be associated with SLMN, that resemble gingival epulis or giant cell tumor of bone [3].

The clinicopathological features of these nodules and followup of several patients suggest that these lesions are reactive rather than neoplastic [1]. Their exact nature is controversial. These SLMNs are classified as either benign or malignant. Benign mural nodules may appear sarcoma-like or have features of a leiomyoma. These benign nodules are associated with a favorable outcome and must be distinguished from anaplastic carcinoma and true sarcomatous nodules. In our case the nodule had abundant osteoclastic type of giant cells with benign-looking stromal cells.

Sarcomatous nodules usually are larger and are composed of fibrosarcoma, undifferentiated sarcoma, and rhabdomyosarcoma [3]. The nodules of anaplastic carcinoma have microscopic features of nests and sheets of large, round malignant epithelial cells. The SLMNs probably represent reactive and self-limited phenomena within a neoplasia [1]. Their coexpression of vimentin and cytokeratin is consistent with an origin from submesothelial mesenchymal cells which undergo partial transformation into epithelial cells [1]. 

## 4. Conclusion 

This case is reported to highlight the importance of careful and thorough examination of a mural nodule within a mucinous cystic tumor and to ascertain the benign or malignant nature and thus help in appraising the patient regarding its prognosis.
